# Discovery, structural characteristics and evolutionary analyses of functional domains in *Acinetobacter baumannii* phage tail fiber/spike proteins

**DOI:** 10.1186/s12866-025-03790-2

**Published:** 2025-02-12

**Authors:** Shenshen Liu, Tao Lei, Yujing Tan, Xiaoyi Huang, Wenxin Zhao, Huanhuan Zou, Jianhui Su, Ji Zeng, Haiyan Zeng

**Affiliations:** 1 School of Biomedical and Pharmaceutical Sciences, Guangdong University of Technology, Guangzhou, 510006 China; 2 School of Public Health, Xiangnan University, Chenzhou, China

**Keywords:** *Acinetobacter baumannii*, Phage, Tail fiber, Tail spike, Depolymerase, Pectin lyase-like, Pyocin_knob, G3DSA:2.60.40.3940, Receptor binding

## Abstract

**Background:**

The global rise in multidrug-resistant *Acinetobacter baumannii* infections poses a significant healthcare challenge. Bacteriophage offer a promising alternative to antibiotics for treating *A. baumannii* infections. Phage tail fiber and spike proteins are essential for host recognition, with some exhibiting depolymerase activity that aids in degrading the bacterial cell wall, facilitating infection. Detailed studies of the functional domains responsible for depolymerase activity and receptor-binding in phage tail fiber/spike proteins are a crucial step toward developing effective phage treatments.

**Results:**

A total of 32 functional domains were identified across 313 tail fiber and spike proteins from 204 publicly available *Acinetobacter baumannii* phages using InterPro and AlphaFold3. Domains associated with depolymerase function were Pectin lyase-like domain (PLD), phage_tailspike_middle domain (PTMD), Transglycosidases domain (TGD), and SGNH hydrolase domain (SHD). These domains were primarily found in phages from the *Autographiviridae* family, specifically within the *Friunavirus* genus. The predominant PLD domain displayed high variability, with its sequence conserved only in a 25-amino-acid region among two closely related fiber/spike protein lineages. All enzymatic domains exhibit high sequence diversity yet retain structural stability, which is essential for enzymatic function. As for receptor-binding domains, four types of pyocin_knob domains (PKD) were initially identified, characterized by unique β-sheet and α-helix configurations. Each type of PKD exhibited distinct potential receptor-binding sites, primarily located within the α-helix region, and was closely associated with the *Obolenskvirus* genus, as well as the *Autographiviridae* and *Straboviridae* families. The G3DSA:2.60.40.3940 domain, exhibiting minor structural variations, was predominantly found in phages of the *Obolenskvirus* genus. Additionally, a novel Obo-β-sandwich structure, identified as a potential receptor-binding domain, was discovered within *Obolenskvirus* genus cluster. The structural diversity of these receptor-binding domains accounts for their interactions with various receptors.

**Conclusions:**

This research deepens the understanding of the relationship between *A. baumannii* phage genera and the functional domains within their tail fiber/spike proteins, emphasizing the compatibility between structural characteristics and functional roles. The data obtained could serve as a reference for the targeted modification of phages or their tail fiber/spike proteins, enhancing their therapeutic applications.

**Supplementary Information:**

The online version contains supplementary material available at 10.1186/s12866-025-03790-2.

## Background

*Acinetobacter baumannii* is a significant pathogenic bacterium [1]. Known for its ability to strongly adhere to abiotic surfaces, *A. baumannii* frequently causes ventilator-associated pneumonia and other respiratory infections [2, 3]. The treatment of multidrug-resistant *A. baumannii* (MRAB) strains is complicated by antibiotic resistance and rapid bacterial evolution [4], leading to prolonged hospital stays and increased mortality rates [5]. Bacteriophages, viral agents that target and replicate within bacteria, offer a promising alternative to antibiotics for treating *A. baumannii* infections [6]. Unlike broad-spectrum antibiotics, bacteriophages exhibit high specificity toward their target bacteria, enabling precise eradication without disrupting beneficial flora [7]. However, the narrow host spectrum of phages limits their application [8].

Phages exhibit high specificity for their host bacteria due to the precise interaction between phage proteins and receptors on the bacterial cell surface. These receptors may include proteins, lipopolysaccharides (LPS), capsular polysaccharides (CPS), teichoic acids, or other molecules characteristic of the bacterial cell wall or membrane [9]. Many phage proteins directly or indirectly participate in different stages of phage infection [10]. Among these, tail fibers and tail spikes are particularly crucial for successful phage infection, with fibers mediating host recognition and attachment, and spikes aiding in host penetration by degrading the host cell membrane [11]. The specialized domains of these proteins are essential for precise targeting and effective interaction with bacterial hosts [12].

Capsular polysaccharides (CPS), which form the bacterial capsule, act as a vital protective barrier for bacteria. Phages equipped with depolymerases can overcome this defense by specifically recognizing and enzymatically degrading CPS, thereby gaining access to the bacterial cell surface to initiate infection [13]. The extensive structural diversity of CPS, arising from variations in monosaccharide composition, glycosidic linkages, and polymer lengths, gives rise to a wide array of unique capsule architectures [14, 15]. As a result, phages must develop highly specialized depolymerases to recognize and degrade specific CPS structures, thereby limiting their host range to a narrow spectrum of bacterial species [13, 16]. Recent studies have increasingly highlighted the critical role of bacteriophage-encoded depolymerases in the phage-mediated degradation of bacterial capsules, which is essential for facilitating phage infection and offers a promising avenue for developing novel antibacterial therapies [17, 18]. Depolymerases, enzymes produced by bacteriophages that typically constitute a critical component of the phage tail fiber/spike proteins for infecting and lysing bacterial cells, come in two main forms: lyases and hydrolases [19]. In recent studies, Peters et al. identified several functional domains associated with the depolymerase activity of *Acinetobacter* bacteriophage tail fibers from 71 distinct phages [20]. Subsequently, Evseev et al. investigated the relationship between the polysaccharide-degrading enzymes located in the tail spikes of lytic, capsule-specific *Acinetobacter* bacteriophages and the host bacterial CPSs, further advancing our understanding of phage depolymerases [21]. However, the structural characteristics and evolutionary relationships of functional domains associated with depolymerases are still not well understood. Furthermore, LPS, a key component of the Gram-negative bacterial outer membrane, also serves as a receptor for phages, with its structural diversity in the O-antigen region influencing host range and phage binding. However, the identification and structural characterization of functional domains involved in receptor interactions, such as those targeting LPS, remain largely unexplored. Crucially, AlphaFold3 offers unparalleled accuracy in predicting complex biomolecular interactions within a single, unified deep-learning framework [22]. This accuracy extends to predicting structural information for functional domains of phage tail fiber/spike proteins, enabling a comprehensive investigation of all functionally significant domains in both sequence and structure. By integrating these predictions, we can gain a deeper understanding of the molecular basis of specificity, particularly for receptor-binding proteins like tail fibers and tail spikes that facilitate phage attachment to specific bacterial receptors, which is crucial for broadening the applicability of phage therapy.

In this study, we conducted a comprehensive analysis of the tail fiber/spike proteins from 204 *A. baumannii* phages. By constructing a proteomic tree and systematically annotating 313 tail fiber/spike proteins, we identified 32 structural domains and examined their distribution. Integrating sequence analysis with AlphaFold3, we explored the evolutionary and structural characteristics of four functional domains associated with depolymerase activity, uncovering their roles in degrading bacterial capsular polysaccharides. Furthermore, we investigated two receptor-binding domains, revealing their structural diversity and adaptability in bacterial recognition. Our findings offer valuable insights into host recognition and polysaccharide degradation, providing a basis for optimizing phages or their tail fiber/spike proteins to enhance therapeutic potential.

## Materials and methods

### Data collection and statistics analysis

We retrieved all entries from the NCBI nucleotide database using the keyword "*Acinetobacter* phage" excluding partial sequences and retaining only complete genomes. Phage genomes with the host identified as *A. baumannii* were selected. For phage genomes with unclear host information, we consulted relevant literature or contacted genome uploaders to confirm host details. Phage genomes with undetermined host information were excluded. From the NCBI virus database, we set the host condition to "*Acinetobacter*" and downloaded all relevant entries, filtering to retain only complete phage genomes with *A. baumannii* as the host (the collection deadline for all data was May 2024). A comparative check between the NCBI nucleotide database and the virus database was conducted to remove duplicates. The final analysis focused on *A. baumannii* phage genome information, calculating GC content, genome length, and relevant attributes such as isolation source, collection date, and sampling region.

### Phage genome proteomic tree analysis and classification

ViPTree was used to compute sequence similarities between genomes based on tBLASTx and to construct a proteomic tree for all phage genomes [23]. PhaGCN (version 2.0) was employed to predict their family according to the latest ICTV standards, considering only predictions with a confidence level of 1 [24, 25]. Phage classification was validated using NCBI data and PhaGCN analysis. The R package ggtree (version 3.10.1) was utilized to generate a proteomic tree of the whole genomes, annotated with classification results [26].

### Tail fiber/spike protein extraction, phylogenetic tree construction

Pharokka (version 1.7.1) was employed to identify all proteins from phage genomes [27]. Coding sequences (CDS) information from NCBI was reviewed to select proteins related to tail fibers and tail spikes. Since phage tail fiber proteins and spike proteins are often used interchangeably in annotations, NCBI information, and related literature without clear distinction, this study does not differentiate between these two types of proteins and analyzes them together as tail fiber/spike proteins. The tail fiber/spike protein data obtained from Pharokka and NCBI were compared, and for any discrepancies in annotation results, relevant literature was consulted for verification. Proteins that could not be conclusively identified were excluded from this study. FastTree was used to construct maximum likelihood (ML) trees for all tail fiber/spike proteins [28].

### Functional domain annotation and structure characteristic analysis

InterPro was employed to annotate the domains of proteins [29]. Annotation information from different databases for the same functional domain was merged, prioritizing annotations from the CDD, Pfam, and SUPERFAMILY databases. The ML tree of tail fiber/spike proteins, along with domain annotation information, was edited using iTOL [30]. Structural modeling and characteristic analysis of functional domains were conducted using AlphaFold3 and PyMOL [22, 31]. The default 10-iteration optimization of AlphaFold3 was applied, and the top-ranked modeling results with a pTM (Predicted Template Modeling) score greater than 0.5 were utilized.

### Protein 3D structure analysis and molecular docking

AlphaFold3 was employed for the 3D structure prediction of tail fiber/spike proteins and the molecular interactions between protein trimers and calcium ions [22]. Only the top-ranked results with an ipTM (Interface Predicted Template Modeling) score greater than 0.8 were utilized. Molecular interactions between tail fiber/spike proteins and ethylene glycol were analyzed through molecular docking using AutoDock Vina [32]. All structural analyses and visualizations were performed with PyMOL and ChimeraX [31, 33]

## Results

### General Information of Collected Phages

A total of 227 complete phage genomes were collected from the NCBI Nucleotide and Virus datasets. After eliminating duplicates and genomes not associated with *A. baumannii* hosts, we retained 204 complete genomes of *A. baumannii* phages (Table S1). These phage genomes exhibit a broad geographical distribution. The majority are from Russia (n = 47), followed by China (n = 39), while 55 genomes have unknown sampling regions. Regarding the isolation sources, 99 phages were isolated from sewage water, 19 from hospitals, 15 from natural water bodies, and 2 from activated sludge, with the remaining 68 having unknown isolation sources. In terms of isolation dates, except for 47 phages isolated in 2018, the distribution across other years is relatively even (Fig. S1). In the box plot illustrating the distribution of phage genome lengths (n = 204), the lengths are concentrated between 40 and 90 kbp. There is an outlier: phage vB_AbaM_ME3, with a length of 234.9 kbp. The GC content ranges from 35 to 45%, with some outliers, but none exceeding 55% or falling below 30% (Fig. S2).

### Classification and proteomic tree analysis of A. baumannii phages

**Fig. 1:**
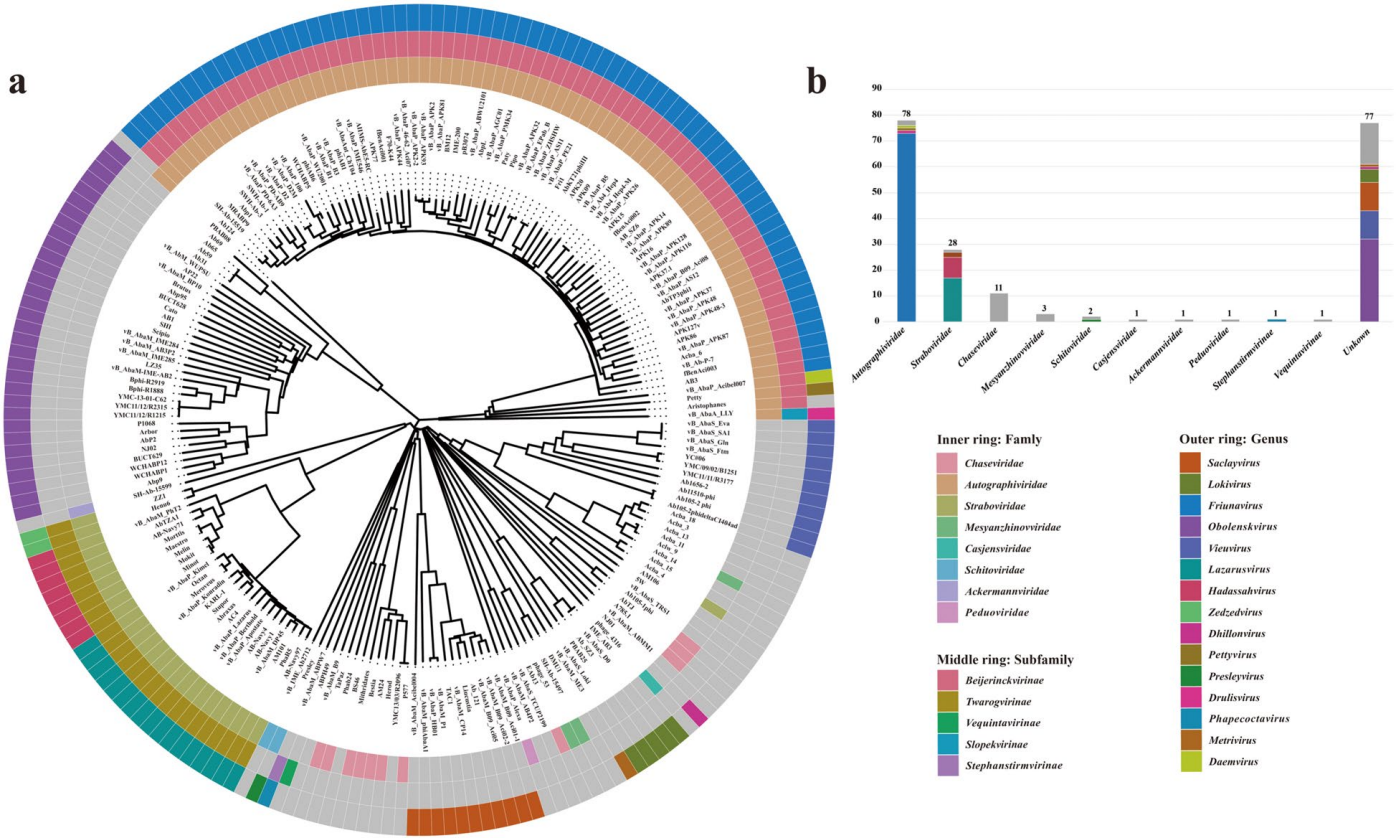
Phylogenetic analysis and classification of 204 *A. baumannii* phages. **a** A circular phylogenetic tree illustrating the classification of 204 *A. baumannii* phages based on their proteomic data. The inner, middle, and outer rings represent family, subfamily, and genus classifications, respectively. Each color corresponds to a different taxonomic group, as detailed in the legend. **b** A bar chart showing the distribution of the 204 *A. baumannii* phages across different families or subfamilies. The number of phages in each family or subfamily is indicated on top of the bars, with color coding consistent with the genus classifications used in the phylogenetic tree

A proteomic tree based on the genome nucleotide sequences of all phages was constructed using ViPTree [23]. Additionally, the classification information for 189 phages (92.6%), determined through both NCBI and PhaGCN software, was integrated into the proteomic tree (Fig. 1a). The classification results reveal that the phage genomes can be broadly categorized into two main families: the *Autographiviridae* family (n = 78, 38%) and the *Straboviridae* family (n = 28, 14%). Within *Autographiviridae*, the predominant genus is *Friunavirus* (n = 73), while *Lazarusvirus* (n = 17) and *Hadassahvirus* (n = 8) are the main genera in the *Straboviridae* family. In addition, some phages that do not belong to any family or subfamily are directly classified at the genus level. These phages primarily belong to three genera: the *Obolenskvirus* genus (n = 32, 16%), the *Saclayvirus* genus (n = 11, 5%), and the *Vieuvirus* genus (n = 11, 5%). The number of phages associated with other families is relatively low, and their genus composition remains unclear (Fig. 1b).

### Distribution of functional domains in phage tail fiber/spike proteins

Using Pharokka, we annotated the complete set of potentially encoded proteins from 204 phage genomes, resulting in 23,906 protein sequences [27]. These proteins were systematically named with the respective phage genome identifier followed by a unique protein sequence number. After cross-referencing this dataset with coding sequence (CDS) information available on NCBI, we selectively retained proteins associated with tail fibers and tail spikes, filtering out those of insufficient length. This curation process yielded a total of 313 tail fiber/spike proteins for subsequent analysis. Notably, the majority of phages (31%) were predicted to encode a single tail fiber/spike protein, while 62 phages (20%) encoded two, and 26 phages (8%) encoded three or four. The number of annotated tail fiber/spike proteins also seems to correlate with phage classification (Table S1).

**Fig. 2:**
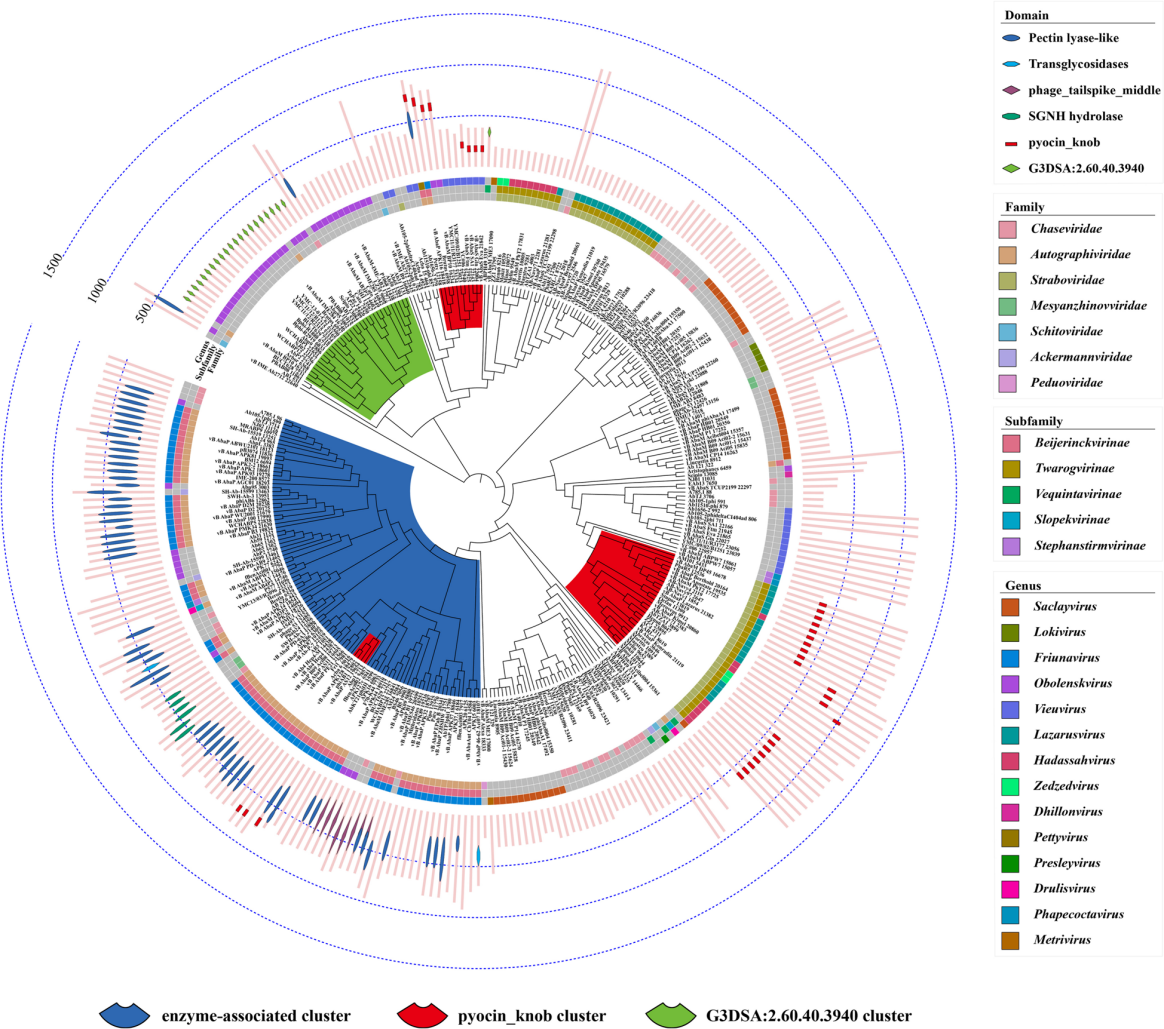
Distribution of six major functional domains in 313 *A. baumannii* phage tail fiber/spike proteins. The ML tree, constructed based on amino acid sequences of 313 *A. baumannii* phage tail fiber/spike proteins, is presented. Bars outside the tree denote the lengths of the tail fiber/spike proteins in amino acids, with identified functional domains indicated by specific shapes and colors. These functional domains are mapped onto the corresponding proteins, with domain types listed in the legend (right panel), including Pectin lyase-like, Transglycosidases, phage_tailspike_middle, SGNH hydrolase, pyocin_knob, and G3DSA:2.60.40.3940. Family, subfamily, and genus classifications of the phages are also indicated with different colors, as explained in the legend. The tree further highlights four distinct clusters based on functional and phylogenetic grouping: enzyme-associated cluster (blue), pyocin_knob cluster (red), and G3DSA:2.60.40.3940 cluster (light green)

After consolidating and refining the annotation results from InterPro, 32 unique structural domains were identified. Some were associated with enzymatic activities, such as the Pectin lyase-like domain and the Transglycosidases domain, etc. Some were linked to receptor-binding functions, including the pyocin_knob domain and the galactose-binding domain-like, etc. And several other domains were identified, such as the Dit-like phage tail protein domain, the Phage Tail Collar domain, the Phage tail fiber repeat domain, the G3DSA:2.60.40.3940 domain, and the G3DSA:2.10.10.20 domain, etc. (Fig. S3 and Table S2). Among these, six major domains, including the Pectin lyase-like domain, phage_tailspike_middle domain, Transglycosidases domain, SGNH hydrolase domain, pyocin_knob domain, and G3DSA:2.60.40.3940 domain, were selected for detailed analysis based on their functional relevance and distribution characteristics. Integrating protein length annotations, classification information, and the distribution of these six domains onto the ML tree revealed that these domains are predominantly located across four major clusters: one enzyme-associated cluster (blue), two pyocin_knob clusters (red), and one G3DSA:2.60.40.3940 cluster (light green) (Fig. 2).

### Domains with enzymatic function

**Fig. 3:**
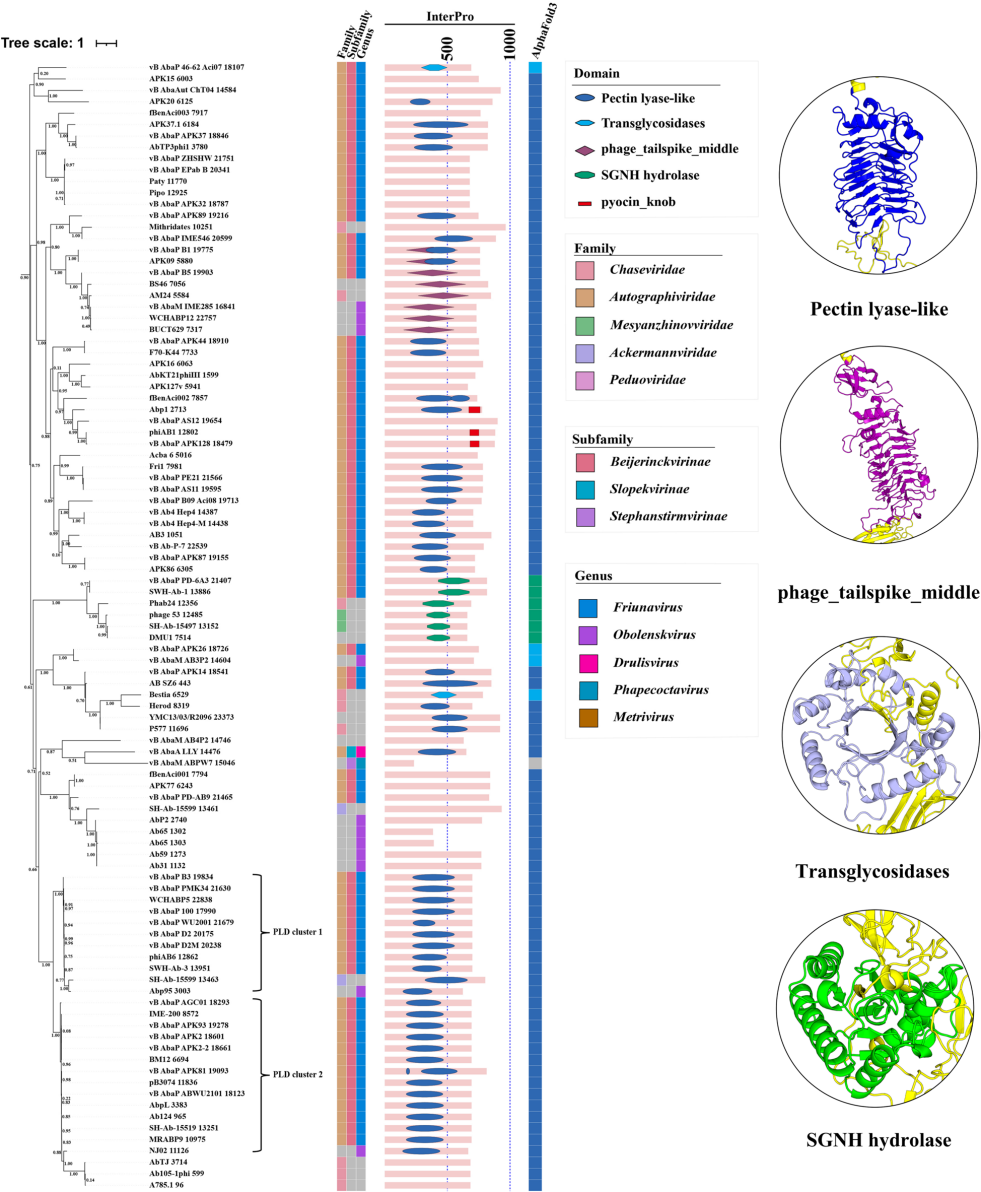
The distribution of domains with enzymatic function in the ML tree. The distribution of key enzymatic domains within the tail fiber/spike proteins, annotated by InterPro, is shown with blue dotted lines marking 500 and 1000 amino acid lengths. Adjacent bar charts represent the AlphaFold3 modeling results. Structural representations of the identified domains are displayed on the right. The Pectin lyase-like domain is depicted in blue, showcasing its right-handed parallel β-helix topology. The phage_tailspike_middle domain is depicted in purple, highlighting its specific structural arrangement. The Transglycosidases domain is depicted in lavender, demonstrating its catalytic structure for hydrolyzing glycosidic bonds. The SGNH hydrolase domain is depicted in green, illustrating its α/β-hydrolase fold, which is crucial in ester bond hydrolysis

Among the identified domains, Pectin lyase-like domain (PLD), phage_tailspike_middle domain (PTMD), Transglycosidases domain (TGD), and SGNH hydrolase domain (SHD) are associated with enzymatic functions. Notably, all four domains are predominantly located within the same major cluster (enzyme-associated cluster, marked in blue on the ML tree; Fig. 2). In this cluster, the middle regions of 65 (67%) tail fiber/spike proteins were annotated by InterPro as enzyme-associated structures. According to AlphaFold3 predictions, with the exception of one protein that is too short to possess any domains, the remaining 96 (99%) sequences contained one of the enzymatic domains: PLD, TGD, or SHD in their middle region (Fig. 3).

### Pectin lyase-like domain (PLD)

Pectin lyases are enzymes that catalyze the cleavage of pectin, a complex polysaccharide found in plant cell walls [34]. These enzymes efficiently cleave the α−1,4-glycosidic bond of pectin molecules through a β-elimination reaction, yielding pectin oligosaccharides [34]. Many pectin lyases exhibit a single-stranded, right-handed parallel β-helix topology. This structure is composed of repeating structural units that stack on top of each other to form the helix. This β-helix topology is a common characteristic of pectinolytic enzymes, including pectin lyases, polygalacturonases, and alginate lyases [35]. The β-helix structure is crucial for substrate binding and catalysis [36]. Using AlphaFold3 modeling analysis, it was demonstrated that all tail fiber/spike proteins annotated as having a PLD display protein structures highly similar to those of pectin lyases, characterized by the single-stranded, right-handed parallel β-helix topology (Fig. 3). The distribution of the PLD domain among the proteins is highly concentrated, with 50 out of 53 proteins (94%) containing the PLD domain clustered together in the ML tree. Notably, 42 (79%) of these proteins belong to the *Autographiviridae* family, specifically the *Friunavirus* genus of bacteriophages. Among the three outliers, one belongs to the *Pettyvirus* genus, also within the *Autographiviridae* family, while the other two belong to the *Obolenskvirus* genus (Fig. 2). This pattern highlights a strong correlation between the presence of the PLD domain and the *Autographiviridae* family.

**Fig. 4:**
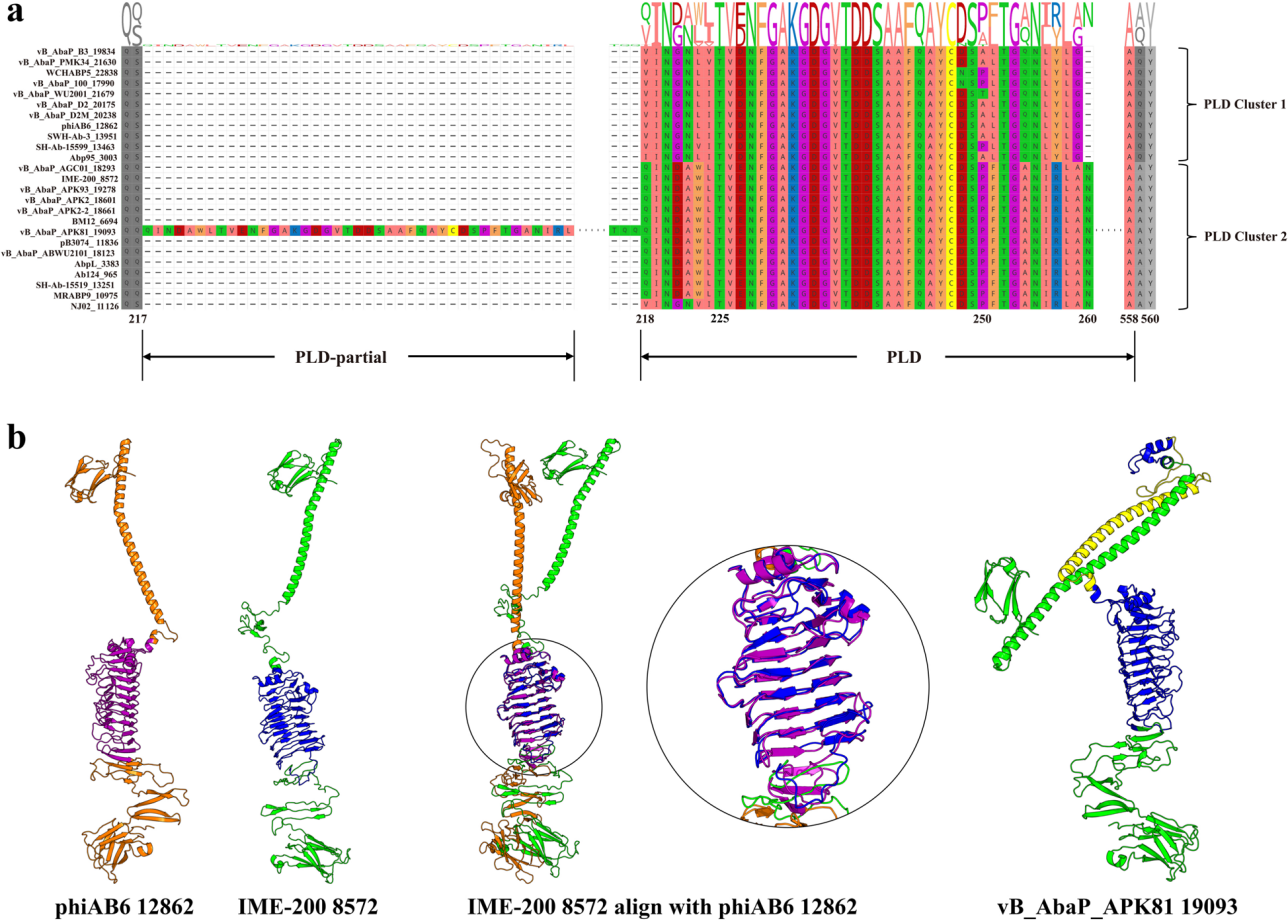
Structural and sequence analysis of PLD-containing tail fiber/spike proteins. **a** Sequence alignment of tail fiber/spike proteins from two PLD clusters, showing that the inserted short sequence between positions 217 and 218 of vB_AbaP_APK81 19,093 is actually part of the entire PLD. **b** Structural models of PLD cluster 1 and cluster 2, including a structural comparison between phiAB6 2862 (PLD cluster 1) and IME-200 8572 (PLD cluster 2), along with a separate detailed view of the vB_AbaP_APK81 19,093 tail fiber protein structure. The PLD regions are depicted in purple and blue, while the inserted structure is shown in yellow

Tail fiber/spike proteins with a PLD domain exhibit extensive genetic diversity, likely driven by variation in CPS, as phages must adapt their tail fiber/spike proteins with specific depolymerase domains to effectively recognize and degrade diverse CPS [16]. Interestingly, the sequences of tail fiber/spike proteins within PLD cluster 1 and PLD cluster 2 are relatively conserved, possibly due to the similar host range of the phages in these clusters. To explore the evolution and variation patterns of PLD, we examined the sequence variation among the two clusters. As shown in Fig. 4a, the PLD amino acid sequences in these clusters were conserved and identical between positions 225 and 249 (numbered according to the phiAB6 12,862 tail fiber protein sequence). These conserved sequences correspond to two short α-helix structures, which serve as critical components linking the long N-terminal α-helix to the central PLD domain’s right-handed parallel β-helix structure. This connection may be essential for maintaining structural integrity of tail fiber/spike proteins. However, divergence gradually increases from the 250th position onwards, forming two evolutionary lineages. Despite slight structural differences due to variations in certain amino acid positions, both clusters exhibited a well-defined right-handed parallel β-helix topology in the PLD regions (Fig. 4b). Interestingly, the vB_AbaP_APK81 19,093 tail fiber protein contains an inserted sequence between amino acid (AA) positions 217 and 218, which closely resembles the N-terminal sequence of the PLD functional domain and is therefore also annotated as PLD. Modeling analysis of this tail fiber protein revealed that the insertion did not disrupt the right-handed parallel β-helix topology of the subsequent PLD region (Fig. 4b).

At the same time, we observed that some tail fiber/spike proteins of bacteriophages previously reported to have depolymerase activity, such as AbTJ 3714, AbP2 2740, and vB_AbaP_AS12 19,654, were not annotated by InterPro as containing PLD or other enzyme-related functional domains [37–39]. However, AlphaFold3 modeling results indicated that they possess β-helix topology structures similar to PLD. Further structural comparison revealed that these β-helix structures, compared to the PLD structures in clusters 1 and 2, have one or more short α-helices or β-strands inserted between the β-helix units (Fig. S4). These inserted sequences disrupt the continuity of the predicted functional domain sequence, which may explain the misannotation by InterPro. However, these secondary structures did not disrupt the right-handed parallel β-helix topology of the functional domain. This suggests that InterPro’s stringent criteria may not always accurately annotate all tail fiber/spike proteins containing such domains, whereas AlphaFold3’s structure-based predictions may provide greater accuracy. In summary, most phages with tail fiber/spike proteins containing this β-helix topology have been confirmed to exhibit depolymerase activity, suggesting that PLD serves as the functional domain of phage depolymerases.

### Phage_tailspike_middle domain (PTMD)

The phage_tailspike_middle domain (PTMD) is composed of two distinct parts: the middle β-helical domain, which resembles the right-handed parallel β-helix structure found in PLD, and a region containing shorter β-strands and α-helices connected by random coils (Fig. 3). Eight tail fiber/spike proteins have been annotated as containing PTMD. Notably, the β-helical structure within the PTMD of APK09 5880 and vB_AbaP_B1 19,775 is also annotated as PLD. Sequence alignment of these eight proteins revealed that the two proteins containing both PTMD and PLD, as well as the six with only PTMD, share some identical sites within the PLD region. Structural modeling of all eight PTMD-containing tail fiber/spike proteins demonstrated that even those not initially recognized as having PLD possess β-helical structures similar to the PLDs in APK09 5880 and vB_AbaP_B1 19,775. However, these proteins feature additional α-helices inserted between the β-helix units, which likely accounts for their lack of PLD annotation by InterPro. Moreover, among the six tail fiber/spike proteins annotated solely with PTMD, those with available information have been confirmed to exhibit depolymerase activity. This suggests that the PTMD domain, including the PLD, is closely associated with depolymerase function.

### Transglycosidases domain (TGD)

Transglycosidases are a category of glycosyl hydrolase enzymes that convert one glycoside into another [40]. In bacteria and fungi, transglycosidases degrade polysaccharides during cell wall turnover, facilitating cell growth, division, and morphogenesis, which are critical for maintaining cell shape and integrity [40, 41]. The Transglycosidases domain (TGD), as annotated by InterPro, shares the same domain as the catalytic domain of transglycosidases and adopts a (β/α)_8-barrel structure, commonly known as the TIM-barrel. This motif, prevalent in the catalytic domains of many transglycosidases, consists of eight β-strands surrounded by eight α-helices, forming a stable and versatile scaffold for catalysis [42] (Fig. 3). Among the 313 phage tail fiber/spike proteins analyzed, only two were annotated by InterPro as containing TGD: vB_AbaP_46-62_Aci07 18,107 and Bestia 6529. However, AlphaFold3 analysis revealed that vB_AbaM_AB3P2 14,604 and vB_AbaP_APK26 18,726 also exhibit a TIM-barrel structure highly similar to TGD. Moreover, previous studies have confirmed that all four phages exhibit depolymerase activity, indicating a close relationship between TGD and depolymerase activity [6, 21, 43, 44].

### SGNH hydrolase domain (SHD)

SGNH hydrolases are a diverse group of enzymes that can break down complex biomolecules, including polysaccharides, lipids, and proteins [45]. In bacteria, SGNH hydrolases are involved in modifying and remodeling the cell wall by degrading polysaccharides into oligosaccharides and monosaccharides [45]. The SGNH hydrolase domain (SHD), as annotated by InterPro, adopts a conserved α/β-hydrolase fold, which consists of a mixed β-sheet flanked by α-helices on either side (Fig. 3). This structural motif is critical for the functional properties of SGNH hydrolases. Six phage tail fiber/spike proteins have been annotated with SHD. Among these, vB_AbaP_PD-6A3 has been reported to possess depolymerase activity, while the depolymerase activity of the remaining five phages is still unknown [46–48]. Given the presence of the SGNH hydrolase domain (SHD) in these phage tail fiber/spike proteins, it is reasonable to speculate that SHD may play a crucial role in conferring depolymerase activity to these proteins.

### Domains with receptor binding function

#### Pyocin_knob domain (PKD)

**Fig. 5:**
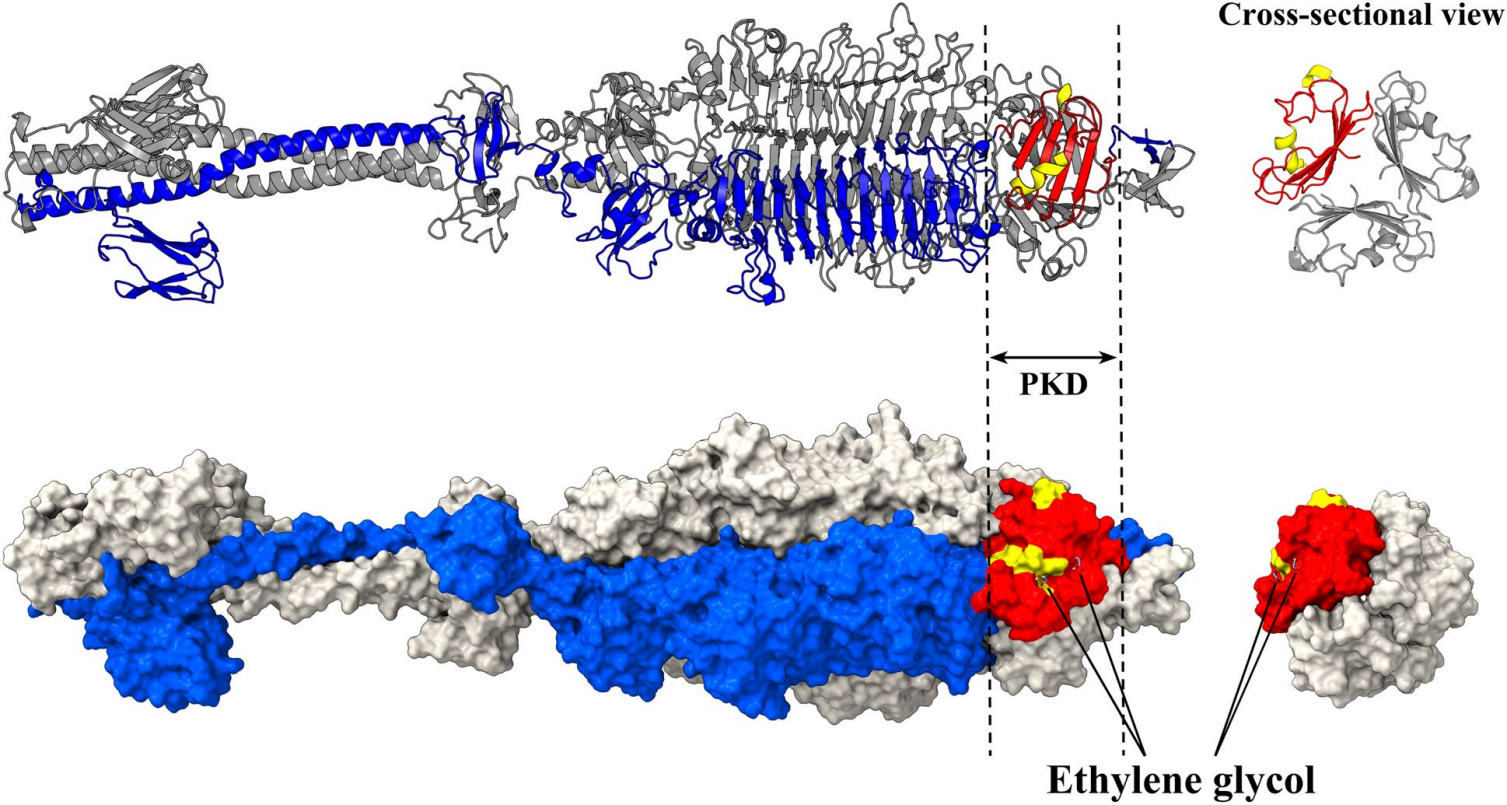
Trimeric structure and surface representation of the PKD-containing tail fiber protein Abp1 2713. The top panel shows the trimeric structure of Abp1 2713, with one monomer highlighted in blue. PKD is characterized by β-sheets (red) buried within the trimeric interface, while α-helices (yellow) and coils form the outer surface. The lower panel presents a corresponding surface representation of Abp1 2713, illustrating the docking of ethylene glycol molecules to the PKD, simulating interactions with bacterial surface components such as lipopolysaccharides (LPS)

R-type pyocins represent a class of bacteriocins synthesized by bacteria within the *Pseudomonas* genus, notably *Pseudomonas aeruginosa* [49]. The functional component of R-type pyocins, known as the knob, plays a pivotal role in recognizing and binding to specific receptors or components on the surface of target bacterial cells [50]. The pyocin_knob domain (PKD) extends beyond pyocins, also featuring in the host-recognition and binding proteins of phages [51]. PKD is characterized by its unique three-dimensional structure, which is crucial for its binding specificity. This domain typically contains multiple β-strands that form a β-sheet structure and may also include α-helical segments that contribute to its overall stability and interaction capabilities [51]. Trimeric modeling of Abp1_2713, which contains PKD, shows that α-helices and random coils form the outer surface of the PKD, while β-sheets are buried in the trimeric interface (Fig. 5). Molecular docking studies have demonstrated that ethylene glycol molecules, which can simulate various parts of lipopolysaccharides (LPS), bind to the α-helices and random coils on the outer surface of the PKD [51]. This suggests that these structural elements play a significant role in the interactive capability and adaptability of PKD, allowing it to fit various bacterial surface receptors (Fig. 5).

**Fig. 6:**
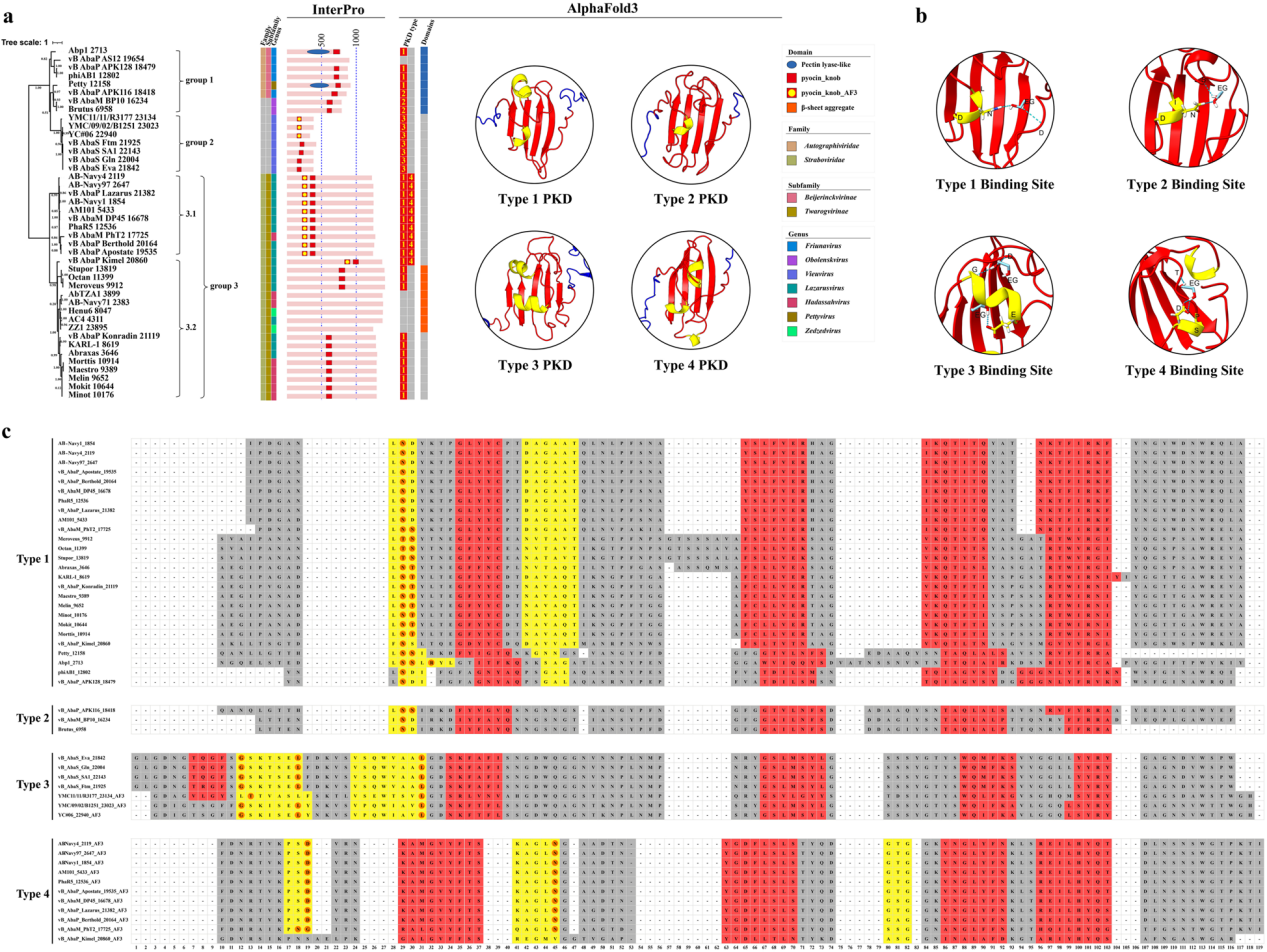
Phylogenetic tree, structural characteristic, molecular docking and sequence analysis of pyocin_knob domain (PKD)-containing tail fiber/spike proteins. **a** Three major groups were identified in the ML tree of tail fiber/spike proteins based on sequence length and structural features. The InterPro panel illustrates the tail fiber/spike proteins’ length, domain annotations, and locations. The AlphaFold3 panel presents PKD types (with numbers corresponding to the type categories) and structural representations of four PKD types, highlighting variations in β-sheet and α-helix arrangements. In the domain legend, pyocin_knob refers to PKD domains annotated by InterPro, while pyocin_knob_AF3 refers to PKD domains predicted by AlphaFold3 but not annotated by InterPro. **b** Molecular docking simulations of four type PKDs were performed with ethylene glycol molecules to evaluate the binding interactions. The side chains of the amino acid residues corresponding to the potential receptor-binding sites on the α-helices are displayed using stick models, with blue dashed lines representing hydrogen bonds. **c** Sequence alignment results of the four types of PKD regions, where red indicates β-strands, yellow represents α-helices, and orange highlights potential receptor-binding sites

A total of 32 phage tail fiber/spike proteins have been annotated as containing PKDs. These phage tail fiber/spike proteins are distributed across three clusters: two distinct Pyocin_knob clusters and a smaller cluster within the enzyme-associated cluster (all three are highlighted in red on the ML tree, Fig. 2). Extracting the tail fiber/spike proteins from these clusters, we constructed a new ML tree. Based on protein length and PKD distribution characteristics, the annotated tail fiber/spike proteins can be classified into three groups (Fig. 6a).

Group 1 includes eight tail fiber/spike proteins of medium length (500–1000 AAs), of which six belong to the *Autographiviridae* family (five in the *Friunavirus* genus and one in the *Pettyvirus* genus), with the remaining two proteins in the *Obolenskvirus* genus (Fig. 6a). Except for vB_AbaP_AS12 19,654, the remaining seven tail fiber/spike proteins have PKDs located at their C-terminal of the protein, and these PKDs exhibit two distinct structural types. Type 1 PKD (n = 4) is characterized by a β-sheet formed by four β-strands, with a short α-helix inserted before the first β-strand and another α-helix inserted between the first and second β-strands. Type 2 PKD (n = 3) is characterized by a short α-helix followed by a β-sheet formed by four β-strands (Fig. 6b). Mutations at specific amino acid sites in vB_AbaP_AS12 19,654 result in a β-sheet structure without any α-helices, potentially explaining the loss of its PKD domain. Additionally, AlphaFold3 predictions show that all tail fiber/spike proteins in this group contain PLDs in their middle regions, and nearly all of them have been reported to exhibit depolymerase activity [17, 20, 52].

Group 2 includes seven short tail fiber/spike proteins, each around 400 AAs in length, all belonging to the *Vieuvirus* genus (Fig. 6a). Among these, four tail fiber/spike proteins have been identified with PKDs located in the middle regions, as determined by InterPro. Different from the PKDs in group 1, PKDs in group 2 are type 3 PKD, characterized by a β-sheet formed by five β-strands, with two parallel α-helices inserted between the first and second β-strands (Fig. 6a). The structures of the remaining three proteins (YC#06 22,940, YMC11-11-R3177 23,134, YMC-09–02-B1251 23,023) revealed that they also exhibit type 3 PKDs in middle regions. Interestingly, several amino acid variations were observed in the region of the first β-strand (Fig. 6c). In YC#06 22,940 and YMC-09–02-B1251 23,023, the amino acid at position 8 (relative to the 108th residue of vB_AbaS_Eva 21,842) mutated from glutamine to serine, resulting in the loss of the first β-strand. In YMC11-11-R3177 23,134, mutations occurred at positions 7, 8, and 10. Despite these mutations, the amino acids at positions 7 to 10 still form the first β-strand of type 3 PKD.

Group 3 comprises 27 phage long tail fiber/spike proteins, ranging in length from 1200 to 1500 AAs. These proteins all belong to the *Straboviridae* family, *Twarogvirinae* subfamily. Among them, 17 (63%) belong to the *Lazarusvirus* genus, 8 (30%) belong to the *Hadassahvirus* genus, and the remaining two (7%) belong to *Zedzedvirus* genus. In this group, 22 (81%) tail fiber/spike proteins were annotated with PKDs by InterPro, and structural models indicate that all of these are type 1 PKDs. Due to divergence in the phylogenetic tree, this group can be further divided into two sub-groups, group 3.1 and group 3.2, based on evolutionary relationships (Fig. 6a). Group 3.1 contains 10 tail fiber/spike proteins, all of which have type 1 PKD located around 320–410 AAs. Interestingly, we identified a new type of PKD, named type 4 PKD, located around 210–300 AAs. Compared to type 1 PKD, type 4 PKD has an additional short α-helix inserted between the second and third β-strands of its β-sheet (Fig. 6a). In Group 3.2, which consists of 17 tail fiber/spike proteins, 12 have PKD domains situated in the middle region of the protein sequences, specifically between 500–1100 AAs. Notably, vB_AbaP_Kimel 20,860 diverges significantly in sequence compared to the other 11 tail fiber/spike proteins. In this protein, a type 1 PKD domain is located between 955–1034 AAs, placing it closer to the C-terminal than is typical for this group. Additionally, it contains a type 4 PKD domain between 820–920 AAs. Several mutations were observed in the type 4 PKD of vB_AbaP_Kimel 20,860, including an aspartic acid-to-serine substitution at position 19, which results in the loss of the first α-helix (Fig. 6c). Interestingly, five tail fiber/spike proteins that lack the α/β architecture typically associated with PKD, along with three proteins containing type 1 PKD domains, all feature a β-sheet aggregate (approximately 260 AAs in length) in their central regions (Fig. 6a and S6b). The potential receptor-binding function of this novel structure remains to be elucidated in future studies.

The position of the PKD domain on tail fiber/spike proteins varies slightly across different groups. In Group 1, the PKD domain is located at the C-terminus of the protein, immediately following the PLD domain. In contrast, in Group 2 and Group 3.2 (except vB_AbaP_Kimel 20,860), the PKD domain is positioned in the middle of the protein. In Group 3.1, Type 1 PKD and Type 4 PKD are arranged in tandem near the N-terminus, separated by approximately 20 AAs. Proteins with PKD domains located at the C-terminus may employ a more straightforward interaction mechanism, while those with PKD domains positioned in the middle or in tandem near the N-terminus may facilitate more intricate receptor-binding processes. However, how this positional variance impacts the functional role of the PKD domain remains an intriguing question, warranting further experimental validation in future studies.

Based on the above analysis, we have identified four types of PKD, each closely associated with specific genera of phages (Fig. 6a). Despite minor variations in the β-sheet and α-helix configurations across the different PKD types, molecular docking analysis demonstrated that all PKD types can bind to ethylene glycol (Fig. 6b). In type 1 and type 2 PKD, it is mainly the asparagine (N) and threonine (T) of the first α-helix that form hydrogen bonds with the ethylene glycol molecule. In type 3 PKD, it is primarily the glycine (G) and leucine (L) of the first α-helix and the leucine (L) of the second α-helix that form hydrogen bonds with the ethylene glycol molecule. In type 4 PKD, it is mainly the aspartic acid (D) of the first α-helix and the asparagine (N) of the second α-helix that form hydrogen bonds with the ethylene glycol molecule (Fig. 6b and 6c). These findings suggest that the receptor-binding site of PKD is primarily localized at the amino acid residues within the α-helix, and the random coils between the β-strands also assist in receptor binding (Fig. 6c and S6a). The configuration and amino acid composition of α-helices vary across different PKD types, allowing PKD to adapt to the recognition of a wide range of bacterial surface receptors.

### G3DSA:2.60.40.3940 domain

**Fig. 7:**
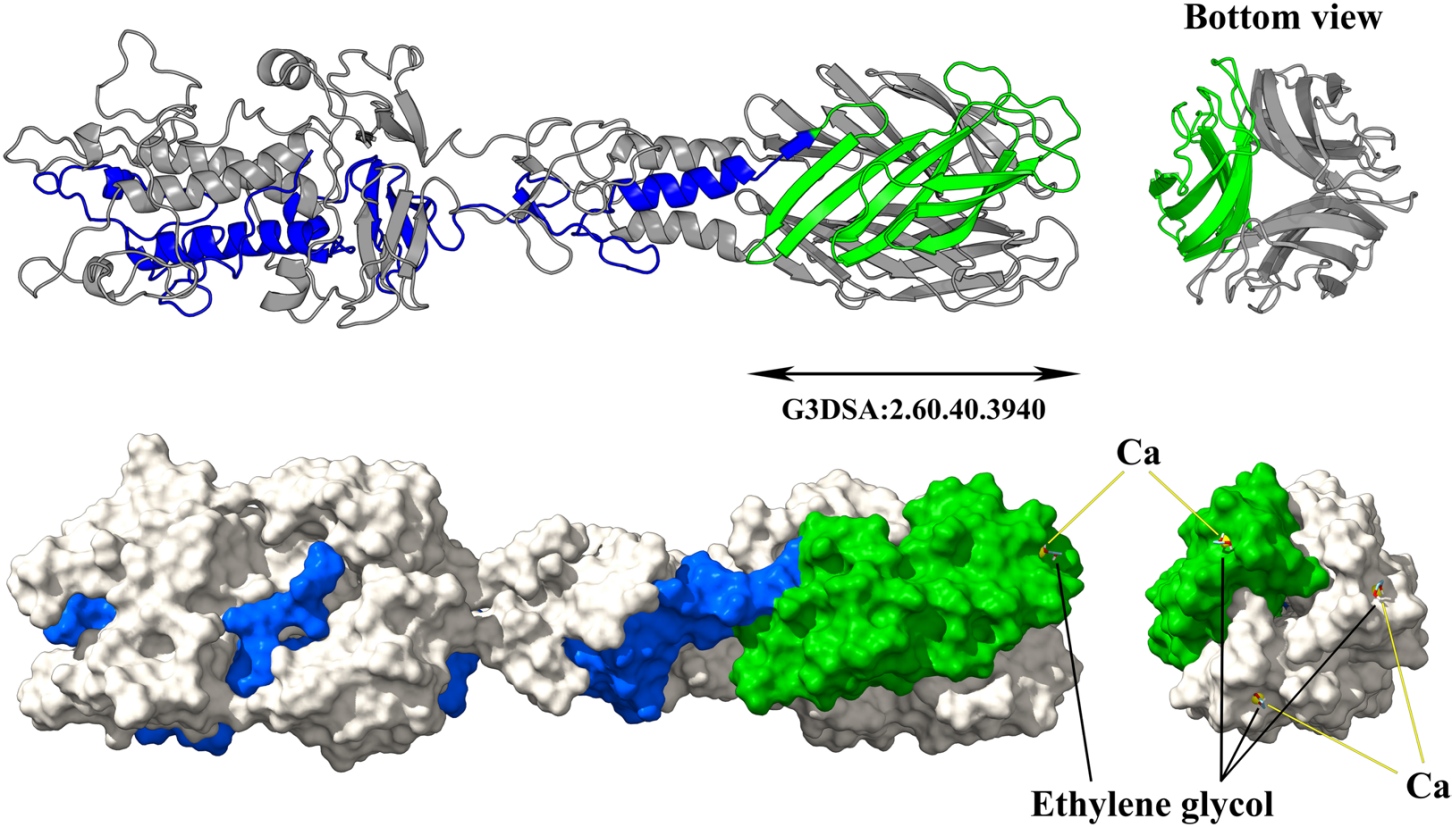
Trimeric structure and surface representation of the G3DSA:2.60.40.3940 domain-containing tail fiber protein vB_Aba_IME284 16,746. The top panel illustrates the trimeric structure of the vB_Aba_IME284 16,746 protein, with one monomer highlighted in blue and the G3DSA:2.60.40.3940 domain highlighted in green. The lower panel provides a surface representation, showing the docking results of the G3DSA:2.60.40.3940 domain with calcium ions and ethylene glycol molecules, which simulates interactions with bacterial surface components such as LPS

The G3DSA:2.60.40.3940 domain, categorized within the Structural Classification of Proteins (SCOP) database, is part of the predominantly beta class (2), featuring a sandwich architecture (2.60) and an immunoglobulin-like topology (2.60.40) within a novel homologous superfamily (2.60.40.3940). Trimeric modeling of the vB_Aba_IME284 16,746 protein, which contains this domain, reveals that it forms a β-sandwich structure at the protein’s C-terminal. The three protein monomers are arranged in a parallel and tightly packed manner, causing the β-sandwiches to aggregate and form a stable columnar structure (Fig. 7). Interestingly, molecular docking studies suggest that the random coils connecting the two β-sheets within each β-sandwich can recognize and bind ethylene glycol and calcium ions in the same region, indicating that the G3DSA:2.60.40.3940 domain may also have a specific recognition function for the LPS on the bacterial surface (Fig. 7).

**Fig. 8:**
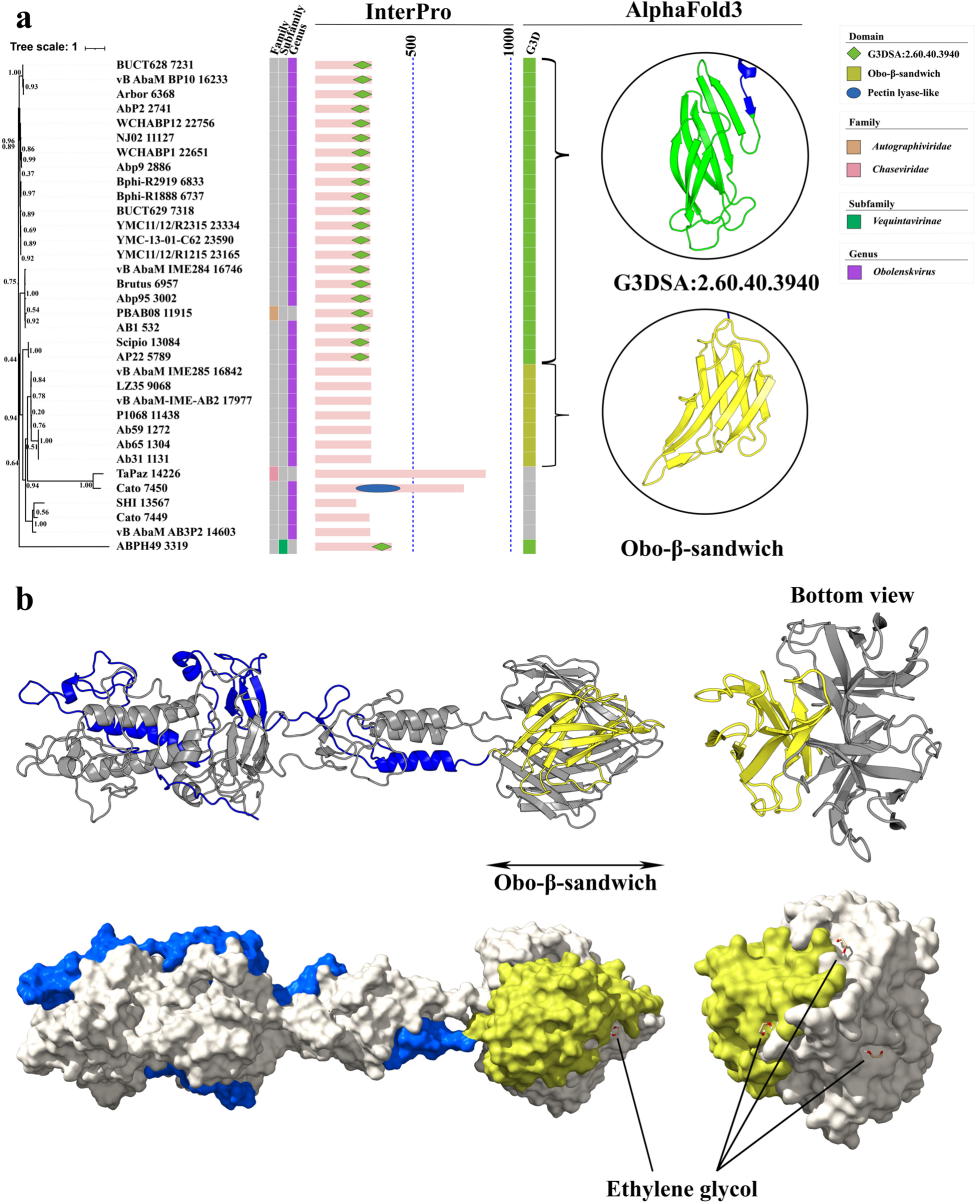
Phylogenetic analysis and structural modeling of phage tail fiber/spike proteins containing the G3DSA:2.60.40.3940 domain. **a** The left panel shows the maximum likelihood (ML) tree based on sequence similarity, with bootstrap values indicated at the nodes. The tree is divided into two groups based on their C-terminal structural characteristics. The middle panel illustrates the domain architecture predicted by InterPro, with the G3DSA:2.60.40.3940 domain highlighted in green at the C-terminal region. The right panel displays structural models of two representative β-sandwich domains generated by AlphaFold3, including the G3DSA:2.60.40.3940 domain and the Obo-β-sandwich. These models reveal variations in β-sandwich structures, showing different configurations of β-strands. **b** The top panel illustrates the trimeric structure of the LZ35 9068 protein, with one monomer highlighted in blue and the Obo-β-sandwich domain highlighted in yellow. The lower panel provides a surface representation, showing the docking results of the Obo-β-sandwich domain with ethylene glycol molecules

There were twenty-two phage tail fiber/spike proteins (around 270–300 amino acids in length) annotated with the G3DSA:2.60.40.3940 domain in the C-terminal region by InterPro. Except for one phage tail fiber protein, ABPH49 3319, the remaining 21 grouped with 14 other tail fiber/spike proteins within the same cluster of the ML tree (G3DSA:2.60.40.3940 cluster, marked in light green on the ML tree; Fig. 2). To further investigate, we extracted all tail fiber/spike proteins from this cluster and added ABPH49 3319 to construct a new maximum likelihood tree (Fig. 8a). Among these, PBAB08 11,915 belongs to the *Autographiviridae* family, while ABPH49 3319 is part of the *Vequintavirinae* subfamily. The remaining 20 proteins belong to the *Obolenskvirus* genus. Structural models of these 22 tail fiber/spike proteins revealed that the G3DSA:2.60.40.3940 domain consists of two compact β-sheets. The first β-sheet is composed of four β-strands, while the second β-sheet contains three β-strands. These seven β-strands are arranged in a folded, interleaved manner, simultaneously forming both β-sheets. The close packing of these β-sheets against each other results in a compact β-sandwich structure (Fig. 8a). Sequence-based analysis revealed that the G3DSA:2.60.40.3940 domains exhibit minor variations, leading to subtle differences in their surface structures. However, these variations do not affect their binding affinity for ethylene glycol and calcium ions (Fig. S7). Interestingly, except for ABPH49 3319, the other 21 tail fiber/spike proteins exhibit highly conserved sequences and structural features in regions outside the G3DSA:2.60.40.3940 domains. In contrast, ABPH49 3319 shows similarity to the other 21 tail fiber/spike proteins only in the G3DSA:2.60.40.3940 domain, with significant differences observed in the rest of its sequence and structure (Fig. S7).

Interestingly, a novel type of β-sandwich structure was discovered in seven tail fiber/spike proteins, each approximately 280 amino acids (AAs) in length, all of which belong to the *Obolenskvirus* genus. Detailed structural analysis revealed that the two β-sheets of this β-sandwich each consist of four β-strands, with a folding pattern distinct from that of G3DSA:2.60.40.3940 domain, resulting in a unique β-sandwich structure at the C-terminal region. Therefore, we have termed this novel structure the Obo-β-sandwich. Trimeric modeling of the Obo-β-sandwich showed that three protein monomers align in a parallel, compact manner, resulting in the aggregation of the β-sandwiches into a stable conical structure, distinct from that of G3DSA:2.60.40.3940 domain (Fig. 8b). Moreover, molecular docking results demonstrated that although the Obo-β-sandwich cannot bind calcium ions, it can still bind ethylene glycol, suggesting that the Obo-β-sandwich domain may have a specific recognition function for lipopolysaccharides (LPS) on the bacterial surface (Fig. 8b).

## Discussion

Phage therapy, as a promising alternative treatment for multi-resistant *A. baumannii* (MRAB), has garnered increasing attention in recent years [4]. A comprehensive understanding of the recognition mechanisms between bacteria and phages is crucial for developing new phage therapies, especially in combating the escalating challenges of multidrug resistance. In this study, we selected 204 *A. baumannii* phages, predominantly belonging to four main genera: the *Friunavirus* genus of the *Autographiviridae* family (38%), the *Hadassahvirus* and *Lazarusvirus* genera of the *Straboviridae* family (14%), and the *Obolenskvirus* genus (16%). A total of 32 unique structural domains were identified, and we further examined the relationships between phage diversity and the localization of functional domains, with particular focus on four representative enzymatic domains (PLD, PTMD, TGD, SHD) and two receptor-binding domains (PKD, G3DSA:2.60.40.3940 domain).

As the most important enzymatic domain, PLD specifically recognizes and digests the bacterial capsular polysaccharide of *A. baumannii* [53]. Sequence alignment shows that PLDs in two similar clusters are conserved from positions 225 to 249 but diverge significantly from position 250 onwards (Fig. 4a). Although the PLD sequences are diverse and highly variable, their structures remain conserved, suggesting that structural stability is crucial for maintaining enzymatic function. However, sequence differences may influence enzyme activity and recognition ability, necessitating further experiments and in-depth analysis to determine the sequence characteristics that optimize enzyme activity. PTMD, which includes the PLD, is also associated with depolymerase activity. TGD exhibits similar activity by catalyzing the hydrolysis of glycosidic bonds, facilitating the degradation of bacterial polysaccharides [54]. SHD is involved in the hydrolysis of ester bonds within polysaccharides and has broad substrate specificity. In phages, SHD also possesses deacetylation activity, potentially contributing to the depolymerization of bacterial capsular polysaccharides [55]. AlphaFold3 modeling revealed distinctive structural features for these domains: the right-handed parallel β-helix topology in PLD and PTMD, the TIM-barrel structure in TGD, and the α/β-hydrolase fold in SHD (Figs. 3 and S5). Collectively, these enzymatic domains highlight the multifaceted strategies employed by phages to degrade bacterial capsular polysaccharides, underscoring their importance in phage infection and their potential for therapeutic applications in targeting bacterial pathogens.

Regarding receptor-binding domains, four types of PKD were identified with distinct β-sheet and α-helix configurations, each with different potential receptor-binding sites located within the α-helix region, highlights the adaptability of PKDs in recognizing diverse bacterial surface receptors. As shown in Fig. 6a, Type 1 PKD, the most prevalent type (n = 26), was found in phylogenetic cluster group 1 and group 3. In group 1, phages with type 1 PKD mainly originated from the *Friunavirus* genus of the *Autographiviridae* family, while in group 3, they were from the *Lazarusvirus* and *Hadassahvirus* genera of the *Straboviridae* family. Type 4 PKDs were also found in phages from the *Lazarusvirus* genus of the *Straboviridae* family in group 3, and they consistently co-occurred with Type 1 PKD. Type 2 PKD, the least abundant type (n = 3), was observed in group 1, associated with the *Friunavirus* genus of the *Autographiviridae* family and the *Obolenskvirus* genus. Type 3 PKD was identified in group 2 tail fiber/spike proteins, all belonging to the *Vieuvirus* genus. These results enhance our understanding of the correlation between PKD types and phage genera, emphasizing the structural adaptability of PKDs in bacterial recognition and their potential applications in phage therapy.

Similarly, the G3DSA:2.60.40.3940 domain also plays a crucial role in receptor recognition through its β-sandwich architecture, which is compact, enabling specific binding interactions [50]. Phages containing the G3DSA:2.60.40.3940 domain are predominantly from the *Obolenskvirus* genus, and the structural variability of this domain is relatively minor compared to the PKD, suggesting a correlation between the structural adaptability of receptor-binding domains and phage genus. Building on the structural characteristics of G3DSA:2.60.40.3940 domain, we discovered another β-sandwich architecture, Obo-β-sandwich, in the same cluster of phages. Compared to G3DSA:2.60.40.3940 domain, the number of β-strands and the way these strands fold to form two β-sheets differ, representing a novel β-sandwich architecture. The presence of G3DSA:2.60.40.3940 domain and Obo-β-sandwich in phage tail fiber/spike proteins underscores their potential functional significance in host recognition mechanisms, supporting the hypothesis that these domains are crucial in the initial stages of phage infection.

This research provides a foundational understanding of the structural and functional diversity of phage tail fiber/spike proteins, offering insights into their roles in host recognition and enzymatic activity. Notably, we find that spatial structures associated with enzymatic functions are relatively conserved, likely due to their necessity for functional stability. In contrast, receptor-binding domains exhibit greater structural diversity, possibly reflecting the need to accommodate a variety of receptors. To deepen our understanding of these roles in phage infection, further experimental validation is needed, particularly concerning the enzymatic activity of various depolymerase-associated domains and the receptor specificity of newly identified PKD types and the Obo-β-sandwich. Integrating high-throughput sequencing and sequence-based analytical approaches with advanced structural modeling techniques, such as AlphaFold3, will enhance the accuracy of domain annotations and predictions.

## Conclusions

In conclusion, this study delves into the species diversity of *A. baumannii* phages and uncovers the structural characteristics and evolutionary traits of functional domains responsible for depolymerase activity and receptor-binding within phage tail fiber/spike proteins. These findings provide key insights into the mechanisms of host recognition and polysaccharide degradation, offering valuable data that can serve as a reference for the targeted modification of phage tail fiber/spike proteins, thereby enhancing their therapeutic potential and paving the way for innovative applications against bacterial pathogens.

## Supplementary Information

Supplementary Material 1: Fig. S1. Geographical distribution, isolation Sources, and collection dates of phage genomes. This figure presents pie charts summarizing the sampling region (a), isolation sources (b), and collection dates (c) of the analyzed phage genomes.

Supplementary Material 2: Fig. S2. Box Plots of Phage Genome Lengths and GC Content in 204 *A.* *baumannii *phages. This figure presents box plots illustrating the distribution of phage genome lengths (a) and GC content (b) across the analyzed samples.

Supplementary Material 3: Fig. S3. Distribution of functional domains in the ML tree of phage tail fiber/spike proteins. This figure illustrates the distribution patterns of 32 unique functional domains across the ML tree of phage tail fiber/spike proteins. Six domains, including the Pectin lyase-like domain, phage_tailspike_middle domain, Transglycosidases domain, SGNH hydrolase domain, pyocin_knob domain, and G3DSA:2.60.40.3940 domain, are highlighted for further analysis.

Supplementary Material 4: Fig. S4. Structural modeling of β-Helix topology in tail fiber/spike proteins not annotated by InterPro as containing PLD. This figure presents the structural modeling of β-helix topology in tail fiber/spike proteins previously reported to have depolymerase activity but not annotated by InterPro (AbTJ 3714, AbP2 2740, vB_AbaM_IME285 16841). The structures reveal short α-helices and β-strands, both highlighted in red, seamlessly integrated into the β-helix units without disrupting their overall topology or functionality.

Supplementary Material 5: Fig. S5. Trimeric surface models of tail fiber/spike proteins containing enzyme-associated domains. This figure presents trimeric surface models of phage tail fiber/spike proteins containing enzyme-associated domains, highlighted with arrows and text annotations. The functional domains depicted include PLD, PTMD, TGD, and SHD.

Supplementary Material 6: Fig. S6. Trimeric surface models of PKDs and β-sheet aggregate. (a) The upper panel presents trimeric surface models of phage tail fiber/spike proteins with identified PKD types (1-4) clearly marked and annotated. In the lower panel, the molecular surfaces of type 2-4 PKDs (type 1 PKD was shown and documented in Fig. 5) are displayed during docking with ethylene glycol, highlighting β-sheets in red and α-helices in yellow. (b) Structural visualization of β-sheet aggregate. The left panel displays both a ribbon view and a surface view of the β-sheet aggregates, marked in orange. The close-up on the right highlights two β-sheets stacked together to form the aggregate.

Supplementary Material 7: Fig. S7. Structural analysis of the G3DSA:2.60.40.3940 domain of AbP2 2741, vB_AbaM_BP10 16233, and ABPH49 3319. The figure displays monomeric cartoon and trimeric surface models of AbP2 2741, vB_AbaM_BP10 16233, and ABPH49 3319. Detailed structural characteristics of the G3DSA:2.60.40.3940 domains (highlighted in green) are shown, including bottom views that emphasize the binding sites for ethylene glycol and calcium ions.

Supplementary Material 8: Phage genome sequences.

Supplementary Material 9: Phage tail fiber/spike protein sequences.

Supplementary Material 10: Table S1. Phage genomic features, taxonomy, and tail fiber/spike protein count.

Supplementary Material 11: Table S2. Characteristics and functional domains of 313 tail fiber/spike proteins.

## Data Availability

All data analyzed in this study are included in this published article and its supplementary information files.
