# Supplementary figures and images for: Discovery, structural characteristics and evolutionary analyses of functional domains in Acinetobacter baumannii phage tail fiber/spike proteins
Source: BMC Microbiol. 2025 Feb 12;25:73. doi: 10.1186/s12866-025-03790-2 (PMC11823257; doi:10.1186/s12866-025-03790-2)

**a****Sampling Regions**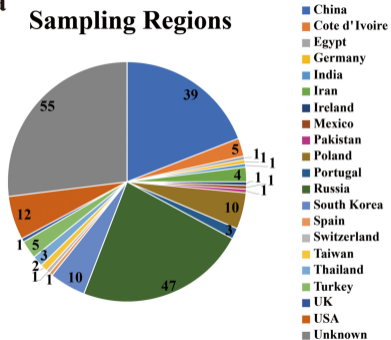**b****Isolation Source**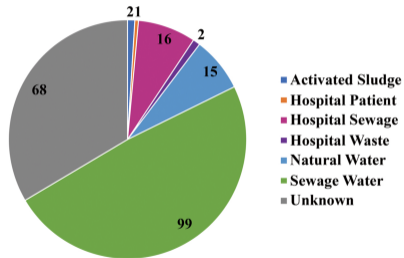**c****Collection Dates**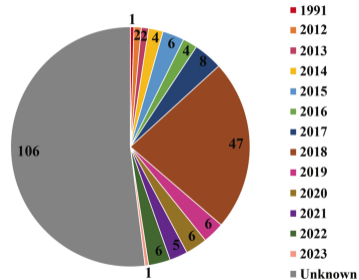

Supplement: Supplementary file 1 — Supplementary Material 1: Fig. S1. Geographical distribution, isolation Sources, and collection dates of phage genomes. This figure presents pie charts summarizing the sampling region (a), isolation sources (b), and collection dates (c) of the analyzed phage genomes. [file 12866_2025_3790_MOESM1_ESM.pdf]

**a**

## Phage Genome Lengths

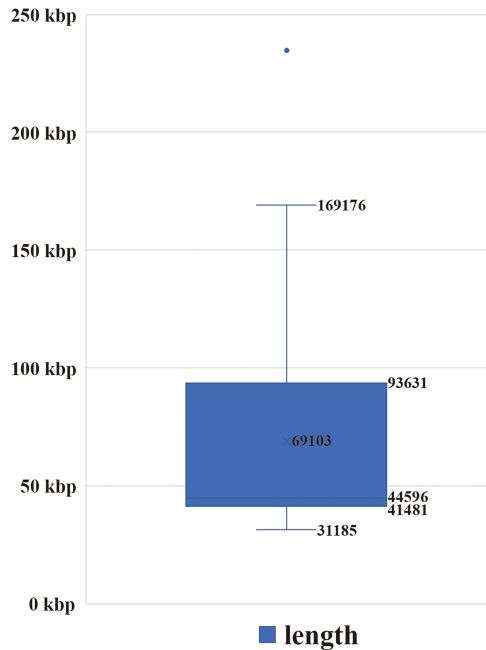**b**

## GC Content

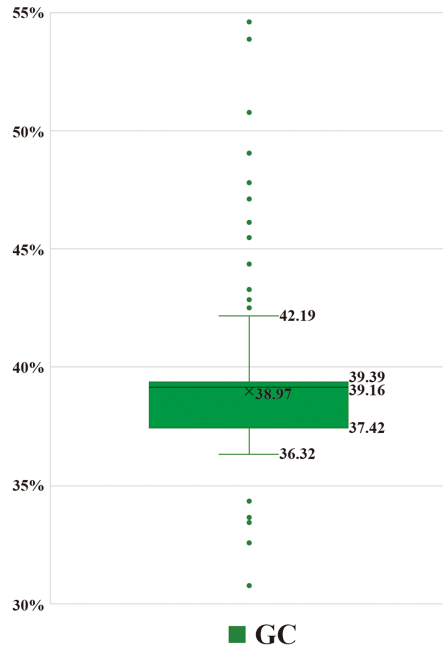

Supplement: Supplementary file 2 — Supplementary Material 2: Fig. S2. Box Plots of Phage Genome Lengths and GC Content in 204 A. baumannii phages. This figure presents box plots illustrating the distribution of phage genome lengths (a) and GC content (b) across the analyzed samples. [file 12866_2025_3790_MOESM2_ESM.pdf]

Tree scale: 1

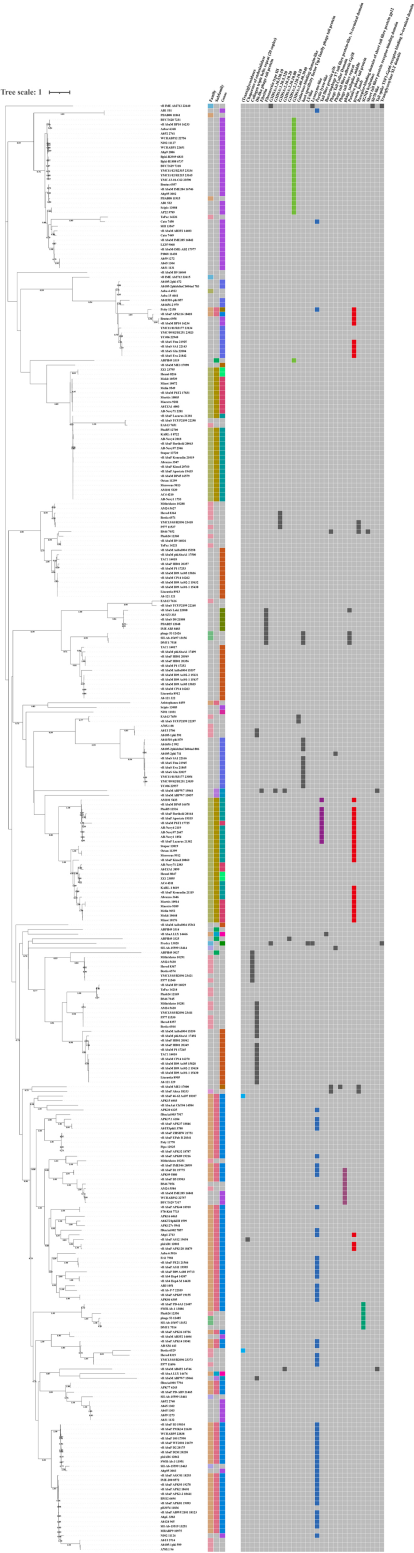

Supplement: Supplementary file 3 — Supplementary Material 3: Fig. S3. Distribution of functional domains in the ML tree of phage tail fiber/spike proteins. This figure illustrates the distribution patterns of 32 unique functional domains across the ML tree of phage tail fiber/spike proteins. Six domains, including the Pectin lyase-like domain, phage_tailspike_middle domain, Transglycosidases domain, SGNH hydrolase domain, pyocin_knob domain, and G3DSA:2.60.40.3940 domain, are highlighted for further analysis. [file 12866_2025_3790_MOESM3_ESM.pdf]

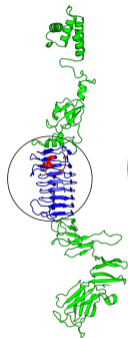

**AbTJ 3714**

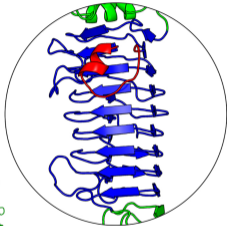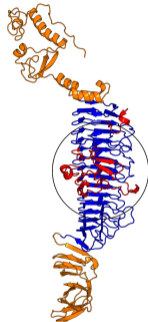

**AbP2 2740**

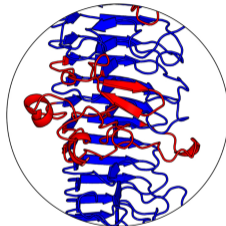

**vB\_AbaM\_IME285 16841**

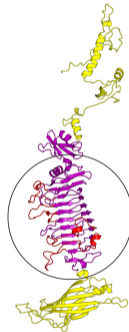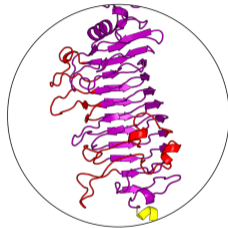

Supplement: Supplementary file 4 — Supplementary Material 4: Fig. S4. Structural modeling of β-Helix topology in tail fiber/spike proteins not annotated by InterPro as containing PLD. This figure presents the structural modeling of β-helix topology in tail fiber/spike proteins previously reported to have depolymerase activity but not annotated by InterPro (AbTJ 3714, AbP2 2740, vB_AbaM_IME285 16841). The structures reveal short α-helices and β-strands, both highlighted in red, seamlessly integrated into the β-helix units without disrupting their overall topology or functionality. [file 12866_2025_3790_MOESM4_ESM.pdf]

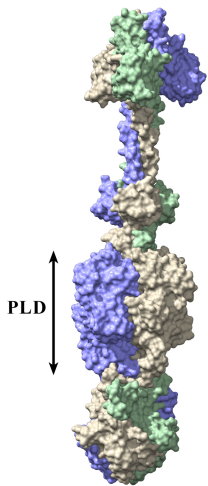

**phiAB6 12862**

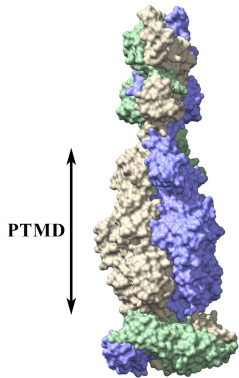

**IME285 16841**

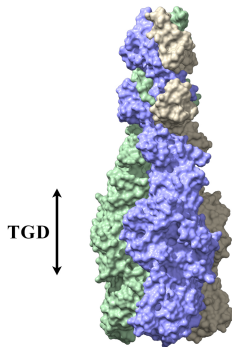

**AB3P2 14604**

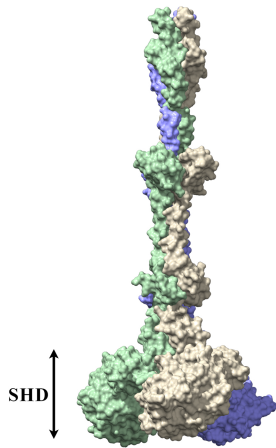

**DMU1 7514**

Supplement: Supplementary file 5 — Supplementary Material 5: Fig. S5. Trimeric surface models of tail fiber/spike proteins containing enzyme-associated domains. This figure presents trimeric surface models of phage tail fiber/spike proteins containing enzyme-associated domains, highlighted with arrows and text annotations. The functional domains depicted include PLD, PTMD, TGD, and SHD. [file 12866_2025_3790_MOESM5_ESM.pdf]

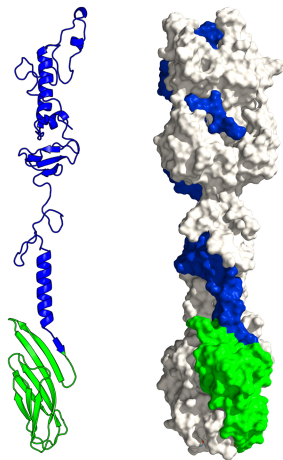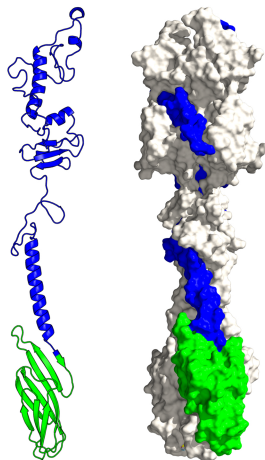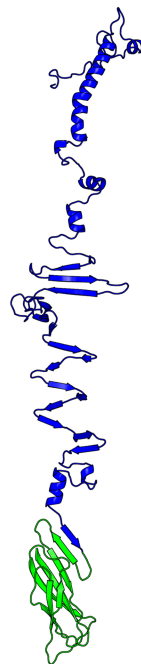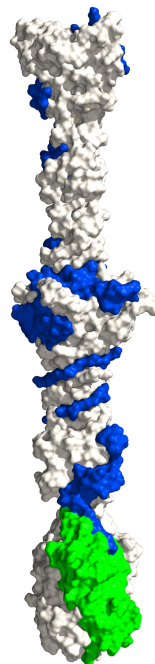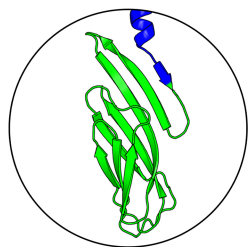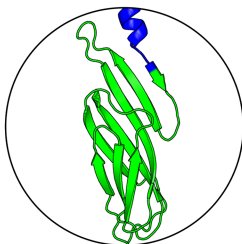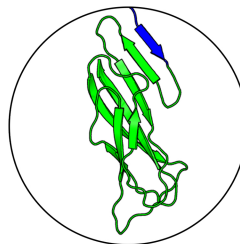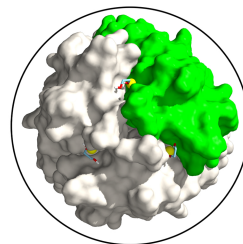

**AbP2 2741**

**vB\_AbaM\_BP10 16233**

**ABPH49 3319**

Supplement: Supplementary file 7 — Supplementary Material 7: Fig. S7. Structural analysis of the G3DSA:2.60.40.3940 domain of AbP2 2741, vB_AbaM_BP10 16233, and ABPH49 3319. The figure displays monomeric cartoon and trimeric surface models of AbP2 2741, vB_AbaM_BP10 16233, and ABPH49 3319. Detailed structural characteristics of the G3DSA:2.60.40.3940 domains (highlighted in green) are shown, including bottom views that emphasize the binding sites for ethylene glycol and calcium ions. [file 12866_2025_3790_MOESM7_ESM.pdf]
