# Supplementary material for: Discovery, structural characteristics and evolutionary analyses of functional domains in Acinetobacter baumannii phage tail fiber/spike proteins
Source: BMC Microbiol. 2025 Feb 12;25:73. doi: 10.1186/s12866-025-03790-2 (PMC11823257; doi:10.1186/s12866-025-03790-2)
Supplement: Supplementary file 6 — Supplementary Material 6: Fig. S6. Trimeric surface models of PKDs and β-sheet aggregate. (a) The upper panel presents trimeric surface models of phage tail fiber/spike proteins with identified PKD types (1-4) clearly marked and annotated. In the lower panel, the molecular surfaces of type 2-4 PKDs (type 1 PKD was shown and documented in Fig. 5) are displayed during docking with ethylene glycol, highlighting β-sheets in red and α-helices in yellow. (b) Structural visualization of β-sheet aggregate. The left panel displays both a ribbon view and a surface view of the β-sheet aggregates, marked in orange. The close-up on the right highlights two β-sheets stacked together to form the aggregate. [file 12866_2025_3790_MOESM6_ESM.pdf]

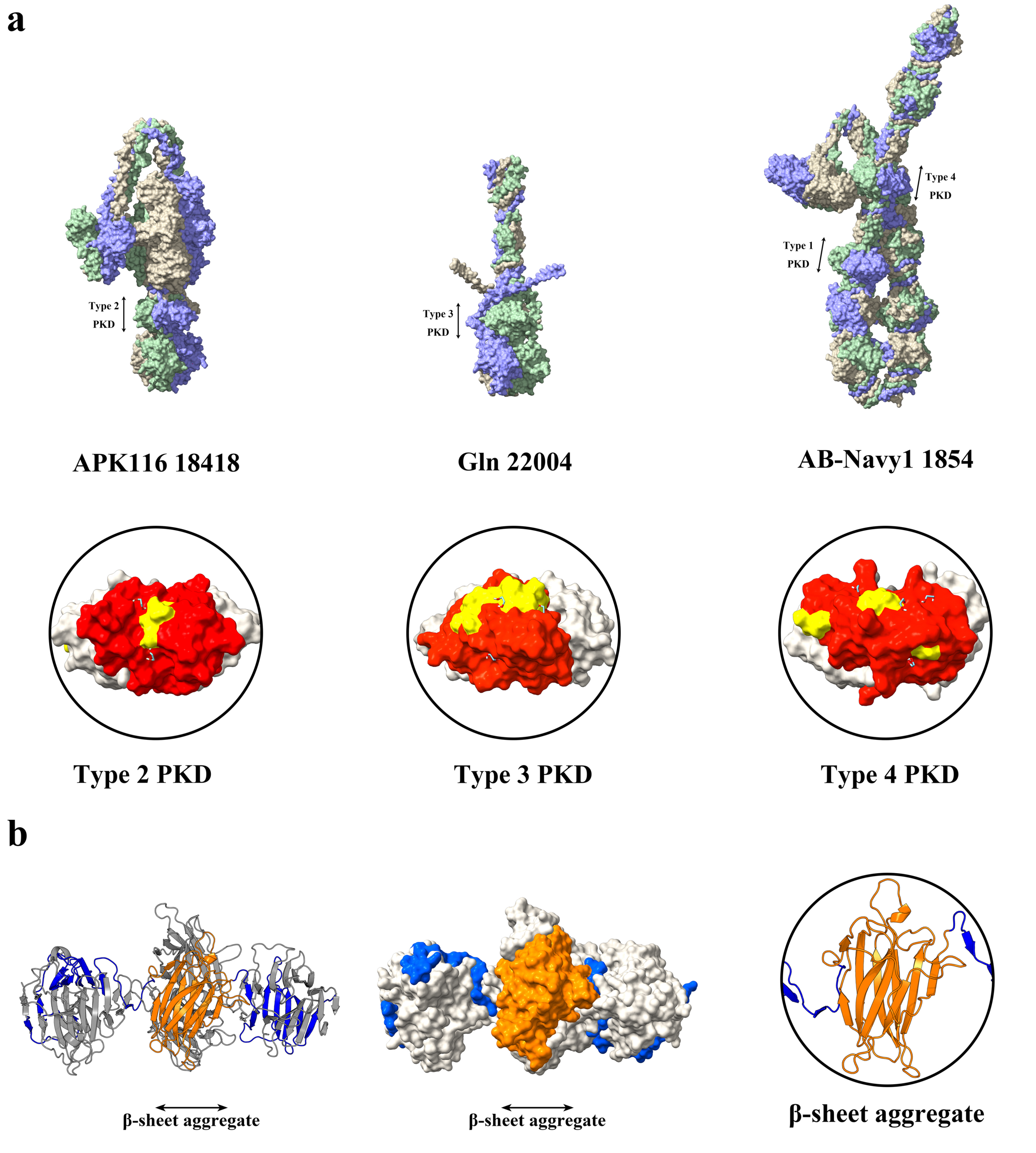
a
Type 2
PKD
Type 3
PKD
Type 4
PKD
Type 1
PKD
APK116 18418
Gln 22004
AB-Navy1 1854
Type 2 PKD
Type 3 PKD
Type 4 PKD
b
β-sheet aggregate
β-sheet aggregate
β-sheet aggregate
